# Cerebral Arterial Thrombosis in Ulcerative Colitis

**DOI:** 10.1155/2013/679147

**Published:** 2013-06-20

**Authors:** Giovanni Casella, Claudio Camillo Cortelezzi, DeLodovici Marialuisa, Princiotta Cariddi Lucia, Verrengia Elena Pinuccia, Vittorio Baldini, Sergio Segato

**Affiliations:** ^1^Medical Department, Desio Hospital, Via Mazzini, 20033 Desio Monza e Brianza, Italy; ^2^Gastroenterology Department, Varese Hospital, Varese, Italy; ^3^Neurology Department, Varese Hospital, Varese, Italy

## Abstract

Thrombosis, mainly venous, is a rare and well-recognized extraintestinal manifestation of inflammatory bowel disease (IBD). We describe a 25-year-old Caucasian man affected by ulcerative colitis and sclerosing cholangitis with an episode of right middle cerebral arterial thrombosis resolved by intraarterial thrombolysis. We perform a brief review of the International Literature.

## 1. Introduction

Thrombosis, mainlyh venous, is a rare and well-recognized extraintestinal manifestation of inflammatory bowel disease (IBD). Arterial Thromboembolic (TE) complications, and in particular strokes, occur less frequently [1]. They usually have embolic origins [2] and represent an important cause of morbidity and mortality. The management of these conditions need a multidisciplinary therapeutic approach and requires awareness for prevention [2]. Here we describe a stroke case due to arterial thrombosis in a patient with ulcerative colitis.

## 2. Case Report

**Figure 1:**
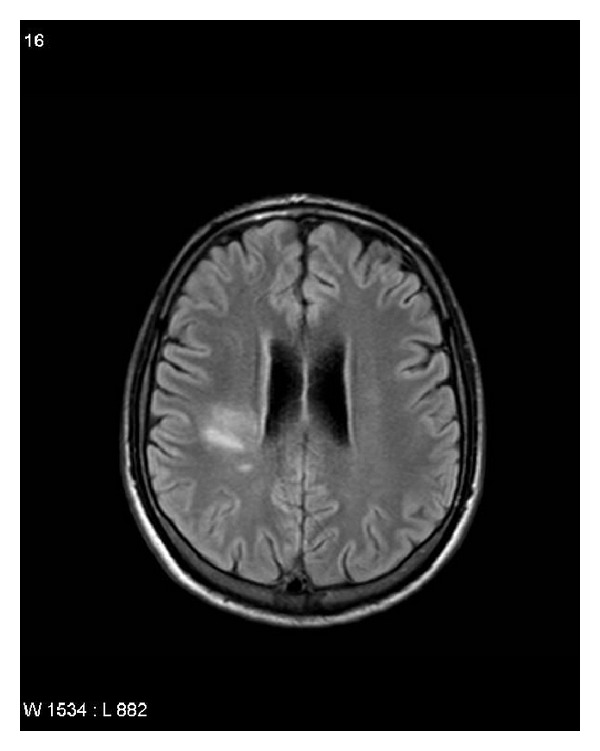
An emergency brain angio-computed tomography (A-CT) evidenced thrombosis of the M2 tract of the right middle cerebral artery.

**Figure 2:**
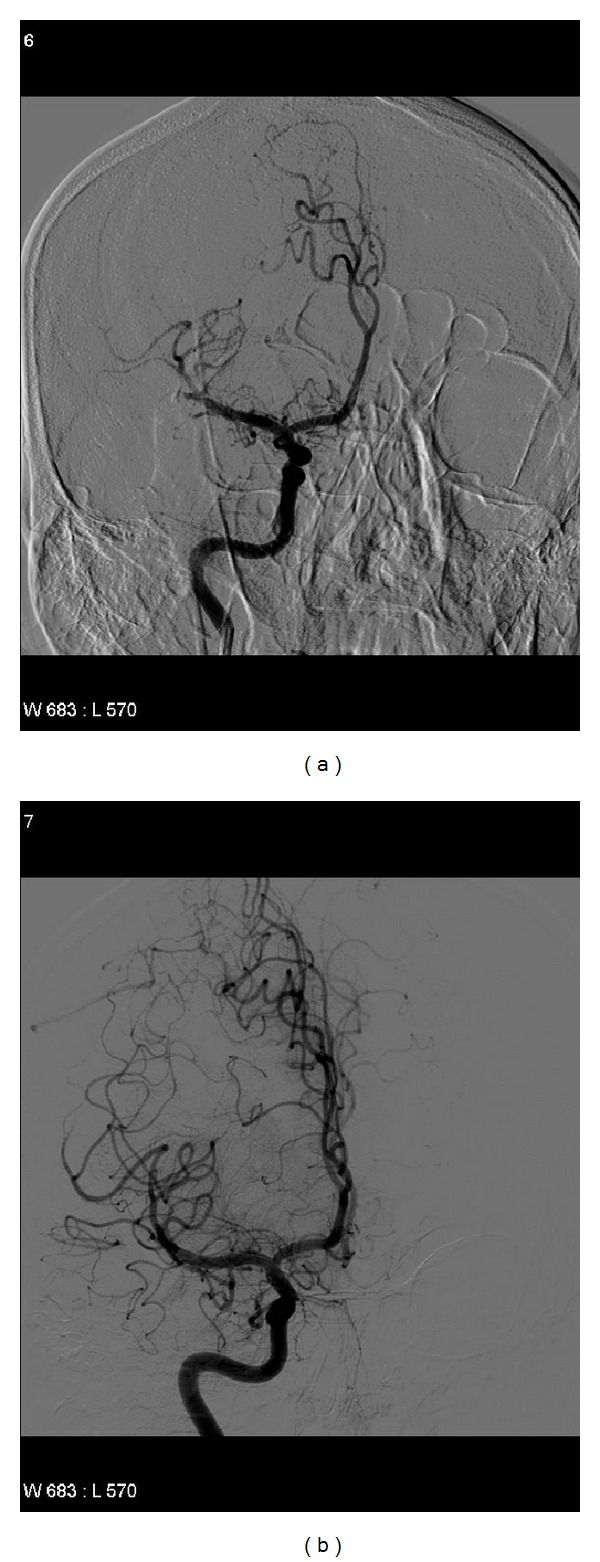
Angiographic appearance of right middle cerebral artery before (a) and after (b) local intraarterial thrombolysis by urokinase (actylise 20 mg).

A 25-year-old Caucasian man was observed in the emergency room for a sudden left-sided hemiparesis characterized by sensitive impairment, confusion and bladder incontinence. NIH stroke scale score (NIHSS) impairment was 8. A diagnosis of ulcerative colitis (UC) and sclerosing cholangitis (SC) had been made 8 years before. He also referred an episode of acute pancreatitis 7 years before as a complication of mesalazine therapy. He had no familial history of cerebral vascular disease (CVD). At clinical examination the patient was normotensive and without fever. Blood tests showed iron deficiency anemia (Hb 7.7 g/dL normal value 13–18), raised white cell counts (WBC 12.470/mmc-n.v. 4000–11000/mmc), erythrocytes sedimentation (ERS) 56 (n.v. < 10), reactive C protein 7.6 mg/L, (n.v.—until 5 mg/dL), gamma Glutamil trans-peptidase (297 U/L-n.v. 11–49), gamma globulin 2.55 g/dL, (n.v. until 1.8 g/dL) antinuclear antibodies 1 : 640 (v.n. negative). An emergency brain angio-computed tomography (A-CT) evidenced thrombosis of the M2 tract of the right middle cerebral artery, without any (apparent) other brain lesions (Figure 1). After an A-CT, an angiographic study was immediately performed, which confirmed thrombosis of the right middle artery, and a local intraarterial thrombolysis by urokinase (actylise 20 mg) was successfully performed (Figures 2(a) and 2(b)). The procedure was complicated by mental alteration with agitation and required a brief observation in an intensive treatment ward recovering within 24 hours (NIHSS 2). The postprocedural brain CT was normal. A following brain nuclear magnetic resonance (NMR) revealed an ischemic lesion in the right middle cerebral artery territory. He had no (familial) risk factors for cerebrovascular disease (CVD). Transthoracic echocardiogram and ultrasound Doppler carotid arteries study were normal. Blood thrombophilic screening (Homocysteine, factor V Leiden, Lupus Anticoagulant Factor, protein C and S, antithrombin) was normal. A diagnosis of Behcet's disease was ruled out because the patient did not evidence genital and/or cutaneous ulcers, and an ophthalmology evaluation did not reveal any alteration. A colonoscopy revealed a picture suggestive for an endoscopic and histological active disease. An abdominal computed tomography (CT) confirmed the irregular dilatation of intra- and extrahepatic biliary tract associated to a “sausage” (bile duct) dilation of 13 mm localized in IV liver segment, a spleen enlargement (bipolar diameter 16 cm) associated to “accessory” spleen of 16 mm in diameter and a little amount of intra-abdominal free fluid. An upper digestive tract endoscopy noted a terminal esophagitis (grade A according to Los Angeles Classification) and erosive duodenitis in the bulb duodenum. The patient, after the discharge, was treated conservatively with Aspirin 100 mg/day, prophylactic doses of low molecular weight heparin (LMWH), immunosuppressive therapy (Azathioprine 150 mg/day), steroid by tapering (Prednisone 50 mg/day), and proton pump inhibitor (Lansoprazole 30 mg/day). At discharge he had a complete normal neurological examination. 

## 3. Discussion

The association of inflammatory bowel disease (IBD) and thrombosis was, for the first time, described in 1936 by Bargen and Baker [13]. Patterson et al. [12], in 1971, described central nervous system (CNS) involvement in a male child with IBD presenting an acute cerebral embolic event. Subjects with IBD have a 3-fold overall increased risk for venous thromboembolism (VTE) compared to controls. The risk of arterial thromboembolic events may be increased in these patients, particularly in those with active disease [3]. Cerebrovascular disorders, in particular, have been documented in 0.12 to 4% of all IBD patients and, perhaps, represent the most described neurological complications [3] in IBD. Often these complications present serious and potentially life-threatening conditions. An increased risk is more evident in patients with active ulcerative colitis and, particularly, in those with ulcerative pancolitis [4]. Even if active disease is associated with an increased risk, some cases have been described during the remission periods [3]. Males and females may be equally affected. The cerebral vascular involvement seems more frequent among younger IBD patients, as reported by Houissa et al., that describes that arterial thrombosis is in 4 patients, 3 of them younger than 25 years [3]. Arterial thrombosis, in particular stroke, may be considered a rare condition [4] (Table 1). Cerebral infarction may be associated to a hypercoagulability condition due to various factors, as qualitative and quantitative abnormalities of platelets, and coagulation factors alterations, as elevation of Factor V, Factor VIII and fibrinogen, deficiency of antithrombin III, and antiphospholipid antibody syndrome [14]. Furthermore Intestinal inflammation may lead to increased risk for thrombosis through several pathways, by activating coagulation cascade, decreasing anticoagulant activity, and inducing hypofibrinolysis, malabsorption, and hypercatabolism with vitamin deficiencies that may lead to hyperhomocysteinemia, a well-known risk factor for thrombosis [3]. Also dehydration, immobility, sepsis, surgery, and corticosteroid therapy determine cerebral thrombosis in IBD patients [14]. The neurological presentation of cerebral arterial thrombosis may be extremely variable, as headache (95%), uni- or bilateral paresis (43%), general or focal seizures (47%) or dysphasia (37%) [3, 4]. The sequel of cerebral vascular thrombosis can be devastating especially in young patients with active and complicated IBD, determining high mortality and disability in about 60% of cases [3]. Conventional computed tomography (CT) or magnetic resonance imaging (RMI) identifies the exact site of cerebral affected areas. At this moment, no guidelines are available for the treatment of cerebral thrombosis and stroke in IBD [4]. Low molecular weight heparin is the most common drug used for the prophylaxis and treatment of vascular thromboembolism [15]. Long-term use of anticoagulant therapy appears safe in the treatment of cerebral sinus venous thrombosis, but clinical experience in treatments of arterial ischemic cerebral lesions is very limited. The presence of hypercoagulable conditions should be always considered an indication for a lifelong anticoagulation with warfarin [15]. Thrombolysis with rTPA, in our case as emergency procedure, was safe and effective; in selected cases mechanical thrombectomy should also be considered. The initial diagnosis of IBD should trigger assessment of thrombosis risk, mainly in young patients with active disease. Rapid evaluation and appropriate multidisciplinary consultation are required for optimal diagnosis and treatments associated to a high awareness in subjects with adjunctive risk factors for thromboembolism as immobility, malignancy, inherited thrombophilia, or surgery.

## Figures and Tables

**Table 1:** “Cerebral Arterial Thrombosis in Ulcerative Colitis”: table of cases reported in international literature.

Author	Sex	Age	Site cerebral ischemia	Extension UC	UC activity	Thrombophilia screening	Drugs	Followup
Houissa et al., (2011) [3]	F	25	Right lent reg and thalamus	Not specified	Yes	Neg	Steroid, LMWH	Recovered
Houissa et al., (2011) [3]	M	24	Left middle cer art	Pancolitis	Yes	Neg	Steroid, LMWH	Residual right hemiparesis
Joshi et al., (2008) [4]	F	24	Left middle cer art	Not specified	Yes	Neg	Azathioprine	Epilepsy
Joshi et al., (2008) [4]	M	55	Right parietal lobe	Left colitis	Yes	Neg	Steroid, LMWH	Colectomy
Keene et al., (2001) [5]	F	13		Not specified	Yes	Not determined		Subtotal colectomy
Fukuhara et al., (1993) [6]	M	18	Ventromedial pons	Not specified	Yes	Not determined	Steroid	Recovered
Tomomasa et al., (1993) [7]	F	12	Left anterior middle cerebral infarction	Not specified	Yes	Not determined	Antibiotics, prednisolone	Seminomatous condition
Paradis et al., (1985) [8]	F	12	Left hemispheric infarct	Not specified	Yes	Not determined		Recovered
Schneiderman et al., (1979) [9]	F	12	Left posterior cerebral artery infarct	Not specified	Yes	Not determined	Steroid, sulphasalazine	Died for cardiac failure
Mayeux and Fahn, (1978) [10]	M	17	Right posterior frontal area infarct	Not specified	Yes	Not determined	Steroid, antibiotics	Slowly improved
Yassinger et al., (1976) [11]	F	15	Right frontal lobe infarct	Not specified	Yes	Not determined	Steroid	Recovered
Patterson et al., (1971) [12]	M	11	Cerebral emboli	Not specified	Yes	Not determined		Colectomy
